# Functional Brain Imaging During Extra-Ocular Light Stimulation in Anophthalmic and Sighted Participants: No Evidence for Extra-Ocular Photosensitive Receptors

**DOI:** 10.3389/fnins.2021.744543

**Published:** 2021-09-28

**Authors:** Holly Bridge, Rupal Morjaria, Stuart N. Peirson, Gaelle S. L. Coullon, Catherine E. Warnaby, Carina A. Pothecary, Brian Leatherbarrow, Russell G. Foster, Susan M. Downes

**Affiliations:** ^1^Wellcome Centre for Integrative Neuroimaging, Nuffield Department of Clinical Neurosciences, University of Oxford, John Radcliffe Hospital, Oxford, United Kingdom; ^2^Oxford Eye Hospital, John Radcliffe Hospital, Oxford, United Kingdom; ^3^Sandwell & West Birmingham Hospitals NHS Trust, Birmingham, United Kingdom; ^4^Nuffield Laboratory of Ophthalmology, Sleep & Circadian Neuroscience Institute (SCNi), University of Oxford, Oxford, United Kingdom; ^5^Central Manchester University Hospitals NHS Trust, Manchester, United Kingdom

**Keywords:** vision, resting state MRI, blindness, non-image forming, functional MRI

## Abstract

Light plays a critical role in regulating physiology and behavior, including both visual and non-visual responses. In mammals, loss of both eyes abolishes all of these responses, demonstrating that the photoreceptors involved are exclusively ocular. By contrast, many non-mammalian species possess extra-ocular photoreceptors located in the pineal complex and deep brain. Whilst there have been suggestions of extra-ocular photoreception in mammals, including man, evidence for these photoreceptors is limited. One approach to objectively determine the presence of such receptors is to measure brain responses to light using functional magnetic resonance imaging (fMRI). Moreover, by using participants who are clinically anophthalmic (congenital and acquired), it is possible to investigate potential light detection in the absence of the retina. Here we scanned participants with anophthalmia and sighted participants in 4 different conditions; the first 3 conditions had a bright light source applied to the following locations: behind the right ear (“ear”), just below the nasal bridge and between the eyes (“head”), and at the right popliteal fossa (“knee”). In the fourth and final scan, the light source was switched off so that there was no light stimulus. All participants were scanned in a completely dark room. No consistent brain activity was detected during any of the light conditions in either sighted controls or anophthalmic participants. Thus, we do not provide any evidence for the presence of extraocular photoreceptors modulating human brain activity, despite recent evidence for gene transcription that may occur as a result of these photoreceptors.

## Introduction

The light/dark cycle produced by the rotation of the Earth provides a dramatic but predictable changing environment under which all life has evolved. As such, light plays a key role in regulating physiology and behavior, including both classical visual responses as well as non-visual responses such as circadian rhythms, hormone synthesis and behavioral responses (Lucas and Foster, 1999; Peirson et al., 2009). In mammals, both visual and non-visual responses are mediated by ocular photoreceptors, and loss of the eye abolishes all responses to light (Foster et al., 1991). In addition to the rods and cones that mediate vision, the mammalian retina contains a third photoreceptive system, based upon the blue-light sensitive photopigment melanopsin. Melanopsin was first identified in Xenopus melanophores (Provencio et al., 1998) and was subsequently shown to be expressed in a subset of retinal ganglion cells in the mammalian retina (Provencio et al., 2000). Further studies showed that these photosensitive retinal ganglion cells (pRGCs) project to the master circadian pacemaker—the suprachiasmatic nuclei (SCN)—to regulate circadian entrainment (Hattar et al., 2002). However, they also project to numerous other central targets and play a key role in many other non-visual responses to light (Hattar et al., 2006; Hughes et al., 2016). By contrast, in non-mammalian vertebrates, numerous extra-ocular photoreceptive sites have been characterized, including the pineal complex, deep brain and dermal photoreceptors (Shand and Foster, 1999; Peirson et al., 2009). These sites typically express specific opsin photopigments, and study of these systems has involved detailed photobiological characterization of the responses, comparing response wavelength sensitivity to the sensitivity of the putative photopigment (Peirson et al., 2009). In humans, extraocular expression of opsins, particularly encephalopsin (OPN3) and neuropsin (OPN5) has been reported in a range of tissues, with high expression in human brain (Halford et al., 2001b; Tarttelin et al., 2003) and skin (Haltaufderhyde et al., 2015; Olinski et al., 2020).

Studies have occasionally reported evidence for extra-ocular photoreception in humans. Twenty years ago Campbell and Murphy (1998) reported that light behind the knee could phase shift both melatonin and body temperature rhythms in humans, and suggested that this may be mediated via humoral phototransduction. However, subsequent studies failed to replicate these findings, which were attributed to confounds in the study protocol, including exposure to low, but biologically active, ocular light levels during extraocular light exposure (Lockley et al., 1998; Hebert et al., 1999; Koorengevel et al., 2001; Wright and Czeisler, 2002; Ruger et al., 2003).

One approach that has been used to determine whether the human brain can respond to non-retinal light stimulation is measurement of the blood-oxygenation-level-dependent (BOLD)-signal using functional magnetic resonance imaging (fMRI). This negates the requirement for visual perception to result from the stimulation. This approach was taken by Starck et al. (2012), who measured resting-state fMRI responses in two groups of people, one who received light stimulation in the ear canal and another who did not. Specifically, they found that there were small differences in a lateral visual network between these two groups, in addition to a difference in the somatosensory cortex. However, there are a number of issues with the approach taken, including the lack of a within-subject design which would have provided a direct comparison between resting state connectivity with and without light. Additionally, the group of participants were scanned several months apart rather than interleaved, which may have led to seasonal differences. Finally, it does not appear that there was any attempt to mask the participants to experimental group by using a light source that was not turned on in the “no light” group. Following this study, there were a number of attempts to use transcranial bright light via the ear canals to treat seasonal affective disorder (SAD) and raise serotonin levels (Timonen et al., 2012; Jurvelin et al., 2014) which was found to be effective. However, the mechanisms by which these approaches are hypothesized to work remain unclear.

In summary, the evidence for the existence of extraocular photoreception in humans remains controversial and weakly supported. Here we investigate the evidence for extraocular photoreception in human subjects who are congenitally and clinically anophthalmic as well as sighted controls. Using functional MRI, we investigate the brain activity in response to extraocular bright light stimulation behind the ear, between the eyes (or sockets) and the infra-popliteal region.

## Materials and Methods

### Participants

Five participants with congenital bilateral anophthalmia (mean age 30 years, range 21–38 years, sex male: female; 4:1) and eight participants with acquired bilateral anophthalmia (mean age 51 years, range 25–70 years, sex male: female; 3:5) participated in this study (see Table 1 for details). Genotyping was available for 2 participants with congenital anophthalmia: case 1 who had a pathogenic variant in OTX2 (Orthodenticle Homeobox 2 gene which plays a critical role in the development of the eyes) and case 6 who had a pathogenic variant in NDP (a region of the X chromosome associated with Norrie’s disease). Seventeen sighted control participants with normal corrected vision also participated (controls had a Snellen best corrected acuity of 6/6 or better in each eye); nine were recruited as controls for the group with congenital anophthalmia (mean age 33 years, range 25–46, male: female; 5:4) and eight as controls for the group with acquired anophthalmia (mean age 41 years, range 33–61 years, male: female; 2:6). The participants with congenital anophthalmia and their sighted controls were scanned in Oxford, whilst the acquired anophthalmic participants and their controls were scanned in Manchester. This study was granted ethical approval by the Local Research Ethics Committee—B/11/SC/0093 and research and development approval from each site. All participants gave informed consent prior to participation, and the study was carried out according to the tenets of the Helsinki agreement. Participant information sheets were provided in Braille for blind participants, and read out verbally or emailed as a word document as per the preferred method of communication requested by the participant.

**TABLE 1:** Demographics and blindness information for all anophthalmic cases.

Subject ID	Sex	Age (years)	Age blindness onset (years)	# years since blind	% of life spent blind
Congenital 1	Male	34	0	34	100%
Congenital 2	Female	38	0	38	100%
Congenital 3	Male	29	0	29	100%
Congenital 4	Male	30	0	30	100%
Congenital 5	Male	21	0	21	100%
Acquired 1	Male	54	2	52	96%
Acquired 2	Male	47	1	46	98%
Acquired 3	Male	71	36	35	49%
Acquired 4	Female	60	40	20	33%
Acquired 5	Female	50	36	14	28%
Acquired 6	Female	25	20	5	20%
Acquired 7	Female	28	21	7	25%
Acquired 8	Female	65	35	30	46%

### Visual Stimulus

Participants were scanned in a very dark room in which the MRI scanner bore lights and the control room lights were off. A blackout blind was additionally used to block light entering the scanner room from the control room. Finally, all participants, both sighted and anophthalmic, wore a blindfold. Participants were requested to lie still and try to relax during four fMRI scans. In three of these scans, a Schott KL2500 cold light source (see Supplementary Figure 1 for spectral power distribution) was used via a 10 m MRI-safe fiber optic light guide. This light source was approximately 56,000 lux (17.3 Log Quanta or 60.47 mW/cm^2^) which is equivalent to daylight on a bright sunny day. The light guide did not produce any sound or heat and participants were explicitly asked whether they could detect heat when the light was on. The participants wore in-ear earplugs during the scan session to attenuate sounds levels from the MRI machine, as required by safety procedures. The light was presented to participants at one of the following locations: behind the right ear (“ear”), just below the nasal bridge and in between the eyes (“head”), and at the right popliteal fossa (“knee”). Justification for each of the locations are i) ear: light had to be shone behind rather than into the ear as the earplugs were essential for protecting hearing, even more important for the participants with anophthalmia ii) head: the light could not be shone directly into the eye socket as this would have been dangerous for sighted participants, and it was important to retain consistency across study participants iii) knee: the effects of light should be equivalent across all participants. The stimulus light was manually turned on for 30 s and then off for 30 s for a total of 8 min at each of these locations. In the fourth scan (“No Light”), the light source was placed by the participant’s right ear but was never turned on. The light intensity was measured using a USB2000 + spectrophotometer and Spectra Suite software (Ocean Insight). Light readings were taken from the following locations: the light source itself, immediately adjacent to the light source, through the ocular prosthesis, and through the blindfold. These readings were performed in a dark room, rather than the MRI scanning suite as the equipment would have been damaged by the magnetic field. No light was detected through the blindfold or through the ocular prosthesis with this method.

### Magnetic Resonance Imaging Acquisition

#### Oxford

Images were acquired using a Siemens Verio 3-Tesla whole body MRI scanner and a 32-channel radiofrequency coil. Structural images were acquired at 1 mm isotropic resolution using a T1-weighted MPRAGE sequence (*TR* = 2,040 ms, *TE* = 4.7 ms, flip angle = 8°, 192 transverse slices). Four functional scan runs were acquired axially using an echo-planar imaging sequence (*TR* = 2,500 ms, *TE* = 30 ms, 3 mm x 3 mm x 2.5 mm voxels, 43 axial slices, 192 volumes).

#### Manchester

Images were acquired using a Philips 3-Tesla whole body MRI scanner and a 32-channel coil. Structural images were acquired at 1 mm isotropic resolution using a T1-weighted MPRAGE sequence (*TR* = 2,439 ms, TE = 8.7 ms, flip angle = 8°, 192 transverse slices). Four functional scan runs were acquired axially using an echo-planar imaging sequence (*TR* = 2,500 ms, TE = 30 ms, 3 mm^3^ isotropic voxels, 36 axial slices, 190 volumes).

### General Linear Model

Functional images were analyzed using tools from FSL (FMRIB’s software library)^1^. Pre-processing included motion correction using MCFLIRT (Jenkinson et al., 2002), brain extraction of motion corrected volumes using BET (Smith, 2002), smoothing with a kernel of 6-mm (full-width at half-maximum), registration to each participant’s T1-weighted structural image using BBR (Boundary Based Registration), and then non-linear registration to MNI-152 standard brain using FNIRT. A high-pass temporal filter of 70 s was used to remove low-frequency fluctuations.

Pre-processed functional images were analyzed using FEAT (FMRI Expert Analysis Tool, version 6.00). Motion correction parameters (translations and rotations in x, y, and z) as well as the time-series of white matter and CSF BOLD signal were included as covariates of no interest in the general linear model (GLM), and thus removed potential motion and physiological noise artifacts from the data. Time-series data were extracted from a 3 mm radius sphere within CSF in the anterior lateral ventricle [MNI coordinates: (2, 10, 8)] and white matter in the dorsal posterior frontal lobe [MNI coordinates: (−26, 22, 28)] (Leech et al., 2012).

The GLM was used to model the 30 s ON/OFF blocks in each experimental condition, and this model was also applied to the scan where no light stimulation was applied.

Group analyses were performed in FEAT using FLAME (FMRIB’s Local Analysis of Mixed Effects) separately for the congenital anophthalmia and acquired anophthalmia populations scanned in Oxford and Manchester, respectively.

### Independent Components Analysis

Functional images were pre-processed and registered using the same parameters described above, except the high-pass temporal filter was changed to 150 s in order to remove frequencies lower than 0.0067 Hz (Watkins et al., 2012). Independent component analysis (ICA) was performed for Oxford and Manchester populations separately using multi-session temporal concatenation in FSL’s MELODIC software (Beckmann and Smith, 2004). This meant that for each site, all pre-processed functional images from all participants (controls and those with congenital or acquired anophthalmia) were temporally concatenated and 25 group-averaged components (or networks of correlated BOLD signal) could be identified from the entire dataset. The following seven classical functional networks were clearly identified for both sites: Default Mode Network, Dorsal Attention Network, Left Fronto-parietal Network, Right Fronto-parietal Network, Visual Network, Auditory Network and Sensorimotor Network.

Next, a dual-regression analysis (Filippini et al., 2009) was used to compare the spatial patterns of these seven networks across the four experimental conditions. Firstly, the time course for each scan and each of the group ICA components was extracted. Secondly, these time courses were used to extract each scan’s spatial map for each of the resting state networks. Finally, these spatial maps were grouped by condition (Ear, Head, Knee, No Light) and voxel-wise testing for significant differences between conditions was performed with FSL’s randomized non-parametric permutation testing (5,000 permutations) using a *t*-test, with correction for multiple comparisons carried out using threshold-free cluster enhanced (TFCE, *P* < 0.05) (Nichols and Holmes, 2002). If no group differences were found in the corrected statistical maps, uncorrected t-statistics were examined to ensure that any subtle differences were not overlooked. Again, populations from the two sites were analyzed separately.

## Results

### Light Did Not Evoke Significant Neural Responses in Any Stimulus Condition

**FIGURE 1:**
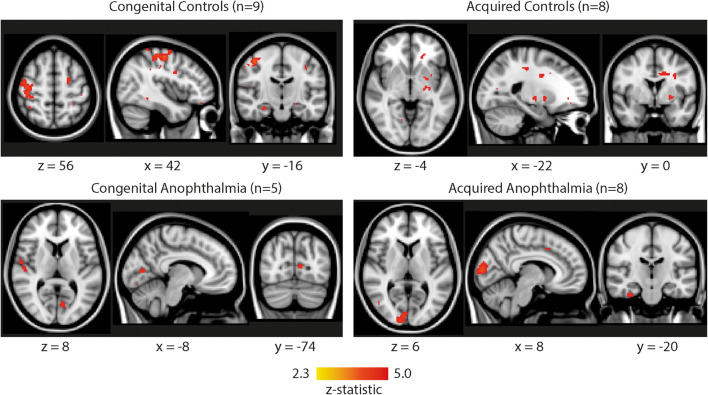
Whole-brain functional activation pooled across all light conditions (ear, head, knee) for each subject group (congenital controls, acquired controls, congenital anophthalmia, acquired anophthalmia). For visualization purposes, statistical maps are thresholded at *Z* > 2.3 (uncorrected) and overlaid on MNI standard brain using FSLview. No results survive cluster correction.

Brain regions showing a significant BOLD response to the 30 s stimulus “light on” condition were identified using a GLM. The same model was also applied to the “No light” condition to control for any inherent modulation at the frequency of light stimulation unrelated to the stimulus. No individual condition generated significant BOLD activity and, even combining across all light conditions (“Ear,” “Head” and “Light”), no brain regions survived cluster correction. Figure 1 shows the uncorrected brain activation patterns combined across all the light conditions. Neither of the sighted control groups showed any consistent activation in the visual cortex, although both the acquired anophthalmia [+8, −92, +6] and congenital anophthalmia [−8,−74, +8] groups showed some BOLD activity in the calcarine sulcus. However, all of these reported clusters are uncorrected with a low threshold of *Z* > 2.3. No clusters survive correction for multiple comparisons.

**FIGURE 2:**
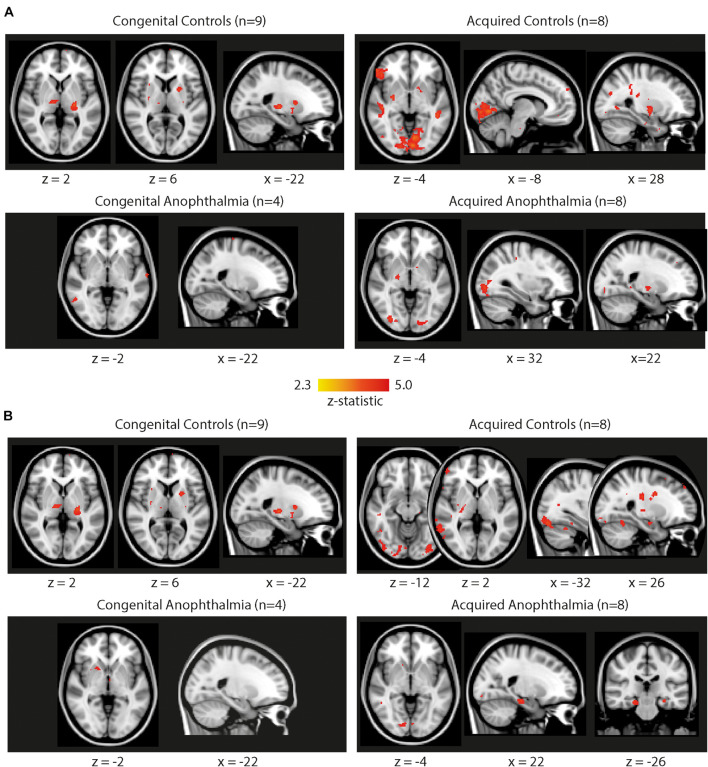
Whole-brain functional activation during the no light condition for each subject group (congenital controls, acquired controls, congenital anophthalmia, acquired anophthalmia). **(A)** Shows results when applying the same 30 s ON/OFF block design as the light conditions in Figure 1. **(B)** Shows results using a 15 s ON/OFF block design. For visualization purposes, statistical maps are thresholded at *Z* > 2.3 (uncorrected) and overlaid on MNI standard brain using FSLview. No results survive cluster correction.

Importantly, similar small clusters appear when applying the same block design to the no light condition. This condition was intended as a control since participants were scanned entirely in darkness with no light stimulation. Figure 2 shows whole-brain results for the no light condition for each of the four subject groups (congenital controls, congenital anophthalmia, acquired controls, acquired anophthalmia), using the same uncorrected low threshold used in Figure 1. Panel A shows results when applying the same 30 ON/OFF model used for the light conditions in Figure 1. Some clusters of “activity” are found in most subject groups, especially across the visual occipital cortex in the acquired control group. Interestingly, when a 15 s ON/OFF model (which does not reflect light and dark periods of the stimulation) was applied to the same No Light dataset (panel B), these clusters of “activity” disappear or appear in different locations in all subject groups. This indicates the spurious nature of any of these “low threshold” regions of activation.

**FIGURE 3:**
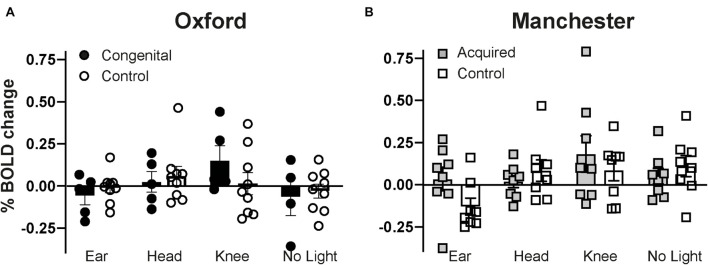
Mean percentage BOLD signal change during each experimental condition (ear, head, knee, no light) extracted from the primary visual cortex (V1, as defined by the Juelich Histological Atlas in FSLview). Group mean values for each of the four experimental conditions are shown in **(A)** for Oxford and **(B)** for Manchester. Error bars represent standard error.

Finally, in order to assess whether group differences (sighted vs. anophthalmia) in the four experimental conditions could be found in a “visual” region of interest, mean percentage BOLD signal change was extracted from the primary visual cortex (V1, defined by the Juelich Histological Atlas as implemented in FSLview and thresholded at 30%). Figure 3 shows the results across the four experimental conditions for each group with congenital anophthalmia, acquired anophthalmia and their respective sighted controls. Individual datapoints, along with group means are shown for each group. V1 percentage BOLD change in the acquired control group during the no light condition matches the cluster of visual cortex activity found at a whole-brain level in Figure 2A. There was no significant effect of either stimulation condition (“ear,” “head,” “knee,” “no light”) group (anophthalmia, control) or interaction indicating little consistent effect of the stimulation in either congenital or acquired anophthalmia.

### No Effects of Light Measured in Data-Driven Approach

To determine whether any networks of brain activity were modulated by the light stimulation, an independent component analysis (ICA) of all functional data (ear, head, knee, no light) was performed for each scan site separately. The ICA revealed the following classical functional networks: default mode network, dorsal attention network, left fronto-parietal network, right fronto-parietal network, visual network, auditory network and sensorimotor network across control and anophthalmia groups.

**FIGURE 4:**
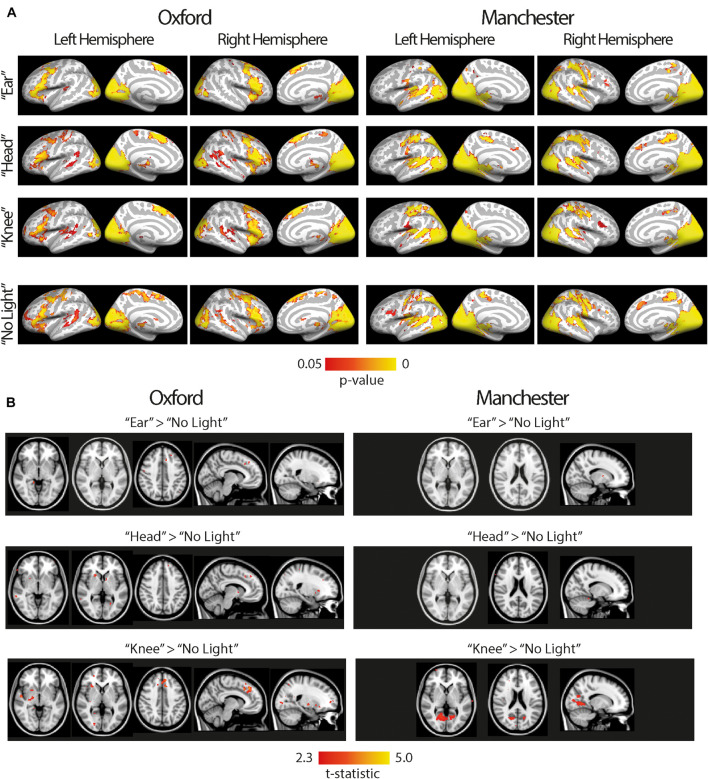
The visual network for all subjects, both anophthalmia and control groups, at Oxford (left) and Manchester (right). **(A)** Shows brain areas that contribute to the visual network in all four experimental conditions (“ear,” “head,” “knee,” “no light”). For visualization purposes, statistical maps are overlaid using Freesurfer onto the inflated cortical surface of the left and right hemisphere average brain. The color bar shows the *p*-value range used to display significant functional connectivity, calculated using permutation testing and threshold-free cluster enhancement (*P* < 0.05). **(B)** Shows a contrast of each light condition (“ear,” “head,” “knee”) > “no light” condition. For visualization purposes, statistical maps are overlaid onto the MNI-152 standard brain using FSLview. Since clusters are small and do not survive cluster correction, uncorrected t-statistics are reported (*T* > 2.3).

A dual regression approach was used to investigate network differences between the four experimental conditions (ear, head, knee, no light), in the visual network previously identified. Figure 4 shows the brain regions that contribute to the visual network in all four experimental conditions (panel A) as well as differences between conditions using contrasts of the three light conditions (ear, head, knee) > no light condition (panel B). All 14 subjects from Oxford (congenital controls and congenital anophthalmia) are shown in the left column and all 16 subjects from Manchester (acquired controls and acquired anophthalmia) are shown in the right column. The brain areas contributing to the network look virtually identical across all four experimental conditions (ear, head, knee and no light). The light > no light contrasts reveal some small clusters in the visual network (Figure 4B). However, none of these clusters survive correction for multiple comparisons and are instead reported as uncorrected *t*-statistics at a very low threshold of *T* > 2.3.

## Discussion

**FIGURE 5:**
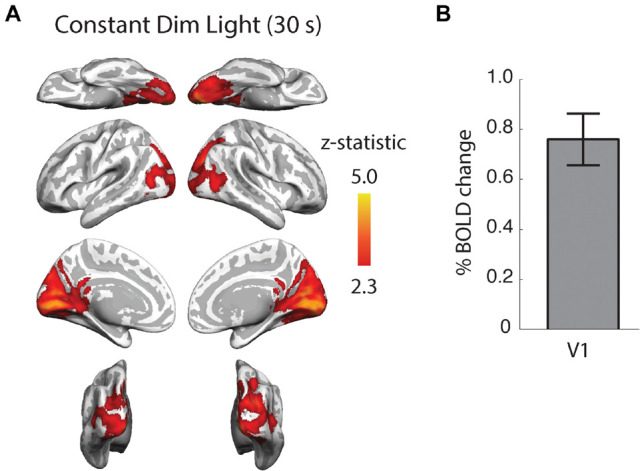
Neural activity in response to constant dim light compared to darkness in 13 healthy, sighted female participants. This weak light stimulus evoked extensive activity across the occipital cortex **(A)** including a significant response in primary visual cortex, V1 **(B)**. Data replotted from Bridge et al. (2015).

We did not find any significant, stimulus-linked brain activity during any of the extra-ocular light conditions; behind the ear, between the eyes or under the knee in congenital and acquired anophthalmia or sighted controls. These data also show that the brain activity does not differ between light conditions in congenital anophthalmia, acquired anophthalmia or control participants, a finding consistent with previous studies (Lockley et al., 1998; Hebert et al., 1999; Ruger et al., 2003). This lack of activity contrasts starkly with the extensive activation seen across the occipital cortex in sighted people even when dim, constant light is presented to the eyes through diffusing glasses. For comparison, Figure 5 shows data from a published study (Bridge et al., 2015) in which the response from 13 sighted participants to this type of dim, diffuse light was measured (A). The signal change in primary visual cortex was large in spite of the lack of image structure, a finding not evident in the current study when considerably higher intensity light was presented to the non-retinal locations.

In the current study, we used an objective measure by reporting the effect of light stimulation at different locations on brain activity. This information was captured using functional MRI scanning, and did not rely on self-reporting by participants (Timonen et al., 2012), which can be subject to bias. Multiple analyses were carried out on the data to distinguish any inherent changes in brain activity from activity evoked by the extraocular light stimulation. Applying a 15 s model instead of a 30 s model produced clusters of activity in several of the experimental conditions even though this period did not correspond to any external light stimulation.

Starck et al. reported increased functional connectivity of the lateral visual cortex on BOLD fMRI imaging when 24 subjects were exposed to extraocular light compared to 26 subjects who received no light stimulation (Starck et al., 2012). However, in addition to issues with the completeness of participant masking, Starck et al. mentioned the sensation of “subjectively perceived change in the visual function” in some subjects which could have resulted from leakage of light activating a visual response (Starck et al., 2012).

The current study employed participants with bilateral congenital and acquired anophthalmia to determine whether any light responses in sighted participants could be due to scattered light reaching the retina. However, neither sighted participants nor participants with anophthalmia showed any consistent response to the extraocular light.

There are a number of potential explanations for the negative result, some of which relate to limitations of the approach, and others to the absence of extra-ocular receptors. Firstly, the study participant numbers are relatively low, so the power to detect an effect across the group is reduced. However, even in cases where diffuse, dim light is detected by the eyes, neural activity is detectable in the occipital cortex for each individual participant (Bridge et al., 2015). In the current study, no individual participant, either sighted or anophthalmic, showed a consistent response to the extraocular light that exceeded the response to “no light.” This single subject approach is unrelated to the size of the population studied. Secondly, the location of the light sources had to be adapted to work in the MRI scanner. The “Ear” stimulus was actually presented behind the ear, rather than in the inner canal. This was a practical solution because participants had to wear ear plugs to protect their hearing in the scanning (particularly important for visually impaired participants). The “Head” stimulus was presented between the two eyes because it was not possible to shine directly into the socket of the anophthalmic participants, and unsafe to shine directly into the eyes of the sighted participants given the brightness of the light source.

A further possibility for a lack of consistent response to the extraocular light is that any extraocular receptors require longer duration stimulation. Light was presented in 30 s periods, as this is a timescale that is amenable to investigation with the BOLD-signal. If, however, the response of extraocular receptors built up over time, this would not be detectable with the protocol used here. Whilst melanopsin pRGCs are capable of responding to short stimulus durations of < 1 s (Do et al., 2009; Adhikari et al., 2015; Joyce et al., 2016) the response kinetics of OPN3 and OPN5 are less well-characterized. Responses associated with these photopigments have often used changes of light/dark cycles or long duration stimuli (> 1 h) (Buhr et al., 2019). Furthermore, the responses described for OPN3 and OPN5 may involve changes in cellular transcription rather than changes in cell membrane potential or firing rates (Buhr et al., 2015, 2019; Nayak et al., 2020; Zhang et al., 2020). If these cellular responses do not result in changes in neuronal activity, this would account for the lack of responses we observe using fMRI.

Previous brain imaging studies (Starck et al., 2012) have suggested that deep brain photoreception may be based upon the expression of the candidate opsin photopigments melanopsin (OPN4) (Provencio et al., 1998), encephalopsin/panopsin (OPN3) (Blackshaw and Snyder, 1999; Halford et al., 2001a) and neuropsin (OPN5) (Tarttelin et al., 2003). In the retina, OPN3 and OPN5 are able to mediate entrainment of the retinal circadian clock (Buhr et al., 2015). Recent work has suggested that OPN5 may also be able to mediate local circadian entrainment in skin (Buhr et al., 2019) as well as hypothalamic preoptic area (Zhang et al., 2020). However, in the retina, when rod, cone and melanopsin signaling is blocked via photoreceptor specific gene knockout, any persisting electrophysiological responses to light appear to occur due to residual responses in a subset of rods which may express cone transducin (Gnat2) (Allen et al., 2010; Hughes et al., 2016). As such, any responses mediated by OPN3 and OPN5 may reflect transcriptional or paracrine changes rather than changes in cell membrane potential or action potential firing. Whilst our negative results in the human brain suggest that brain network activity may be unaffected by light, it cannot preclude subtle long-term effects of extraocular opsin photopigments. Both OPN3 and OPN5 have been suggested to form blue and violet/ultraviolet photopigments, respectively (Yamashita et al., 2010; Kojima et al., 2011; Sugihara et al., 2016). The light source used in the present study certainly contained sufficient short-wavelength light to be able to stimulate such a photopigment, even given attenuation by overlying tissues. Indeed, given the high intensity of light used in the current study, if more light were needed to mediate such deep brain responses, it would seem unlikely that they would play a significant role in natural responses to light. As such, the significance of these extraocular opsins for human physiology and health remains equivocal.

## Data Availability Statement

The raw data supporting the conclusions of this article will be made available by the authors, without undue reservation.

## Ethics Statement

The studies involving human participants were reviewed and approved by the South Central Oxford Research Ethics Committee B/11/SC/0093. The patients/participants provided their written informed consent to participate in this study.

## Author Contributions

HB, RF, SD, and SP designed the research. RM, CW, and CP performed the research. RM, GC, and HB analyzed the research. HB, RM, SP, GC, BL, RF, and SD wrote the manuscript. All authors contributed to the article and approved the submitted version.

## Conflict of Interest

The authors declare that the research was conducted in the absence of any commercial or financial relationships that could be construed as a potential conflict of interest.

## Publisher’s Note

All claims expressed in this article are solely those of the authors and do not necessarily represent those of their affiliated organizations, or those of the publisher, the editors and the reviewers. Any product that may be evaluated in this article, or claim that may be made by its manufacturer, is not guaranteed or endorsed by the publisher.

## Footnotes

1
http://www.fmrib.ox.ac.uk/fsl
