# Supplementary material for: Functional Brain Imaging During Extra-Ocular Light Stimulation in Anophthalmic and Sighted Participants: No Evidence for Extra-Ocular Photosensitive Receptors
Source: Front Neurosci. 2021 Sep 28;15:744543. doi: 10.3389/fnins.2021.744543 (PMC8508779; doi:10.3389/fnins.2021.744543)
Supplement: Supplementary file 1 [file Data_Sheet_1.docx]

Supplementary Figure 1


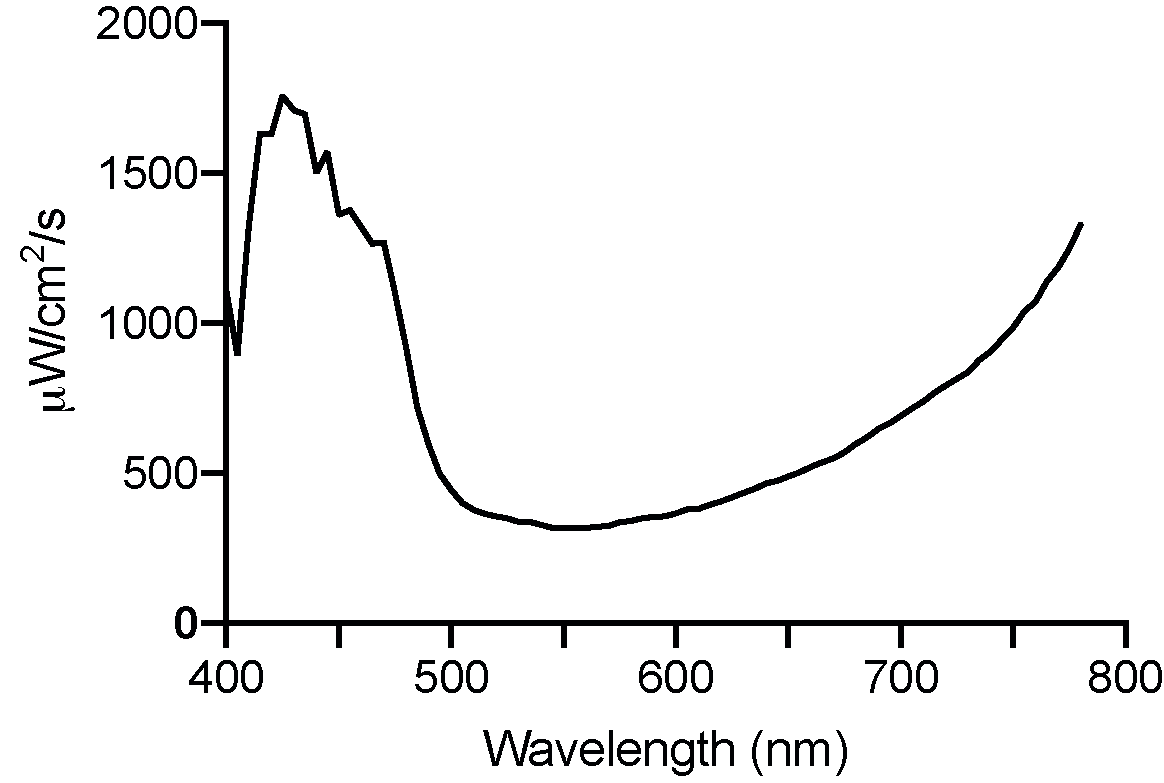


Figure S1: Spectral power distribution of the bright light source by KL2500 cold light source
